# Economic Evaluation of Oral Alendronate Therapy for Osteoporosis in Chinese Postmenopausal Women: The Impact of Medication Compliance and Persistence

**DOI:** 10.3389/fphar.2020.575893

**Published:** 2020-11-16

**Authors:** Ruxu You, Zijie Liu

**Affiliations:** ^1^ Department of Pharmacy, Union Hospital, Tongji Medical College, Huazhong University of Science and Technology, Wuhan, China; ^2^ Institute of Materia Medica, Chinese Academy of Medical Sciences and Peking Union Medical College, Beijing, China

**Keywords:** economic analysis, postmenopausal osteoporosis, alendronate, compliance, persistence

## Abstract

**Objective:** Prevalence of osteoporosis in Chinese postmenopausal women has significantly increased over the past decade and oral bisphosphonates are the most potent antiresorptive drugs. The purpose of the present research was to evaluate the cost-effectiveness of oral alendronate for individuals with osteoporosis. We also assessed the impact of medication compliance and persistence on economic outcomes of alendronate and potential economic evaluations of persistence-enhancing interventions.

**Methods:** We constructed an individual-level state-transition model to project health outcomes and costs of oral alendronate for Chinese postmenopausal osteoporotic women. The impact of medication compliance and persistence on economic evaluation was addressed in various scenario analyses. Model inputs were derived from clinical trials and published sources, where available. Deterministic and probabilistic sensitivity analyses were conducted to explore the impact of uncertainties and assumptions on the cost-effectiveness results.

**Results:** Compared with no treatment, alendronate treatment was associated with an additional 0.052 QALYs (quality-adjusted life-years) at an additional cost of USD 738, which yielded an incremental cost-effectiveness ratio (ICER) of USD 14,192.308/QALY. The ICER for the different scenarios (full compliance, full persistence, and both full persistence and full compliance) was USD 4,933.333/QALY, USD 3,006.849/QALY, and USD 2,049.822/QALY, respectively. One-way sensitivity analysis showed the ICER was most sensitive to variations in time horizon and residual effect. Probabilistic sensitivity analysis demonstrated that, at a willingness to pay of USD 29,340/QALY, the probability that oral alendronate therapy will be cost-effective is approximately 80%.

**Conclusion:** The findings support the view that oral alendronate is cost-effective for the treatment of osteoporotic fractures in Chinese postmenopausal women. Medication persistence is found to have a greater impact on cost-effectiveness than compliance and interventions to improve persistence to be an efficient use of resources.

## Introduction

Osteoporosis, or porous bone, is a disease characterized by low bone mass and structural deterioration of bone tissues, leading to bone fragility and an increased risk of fractures (Watts, 2018). The International Osteoporosis Foundation (IOF) estimates that, by 2050, more than 50% of all osteoporotic fractures will occur in Asia, and China will be most severely affected due to its large population of seniors (Pisani et al., 2016). Fractures significantly affect patients by impairing their ability to perform daily activities. Moreover, a health economics model was developed and forecasted that the costs of osteoporotic fractures in China will double by 2035, rising to approximately USD 25.58 billion by 2050, indicating that, in addition to morbidity and mortality, osteoporotic fractures are also associated with a significant health care expenditure to the society (Si et al., 2015; Liu et al., 2018).

Fortunately, medical advancements have increased the range of therapeutic options available for the prevention and treatment of fractures (Iolascon et al., 2020). Currently, oral bisphosphonates are the most potent antiresorptive drugs for the treatment of osteoporosis in postmenopausal women (Maraka and Kennel, 2015). Multiple meta-analyses and systematic reviews have shown that bisphosphonates are effective in decreasing the risk of various types of bone fractures (Feng et al., 2013; Brown et al., 2014). However, it is widely acknowledged that compliance (the extent to which a patient acts in accordance with the prescribed interval and dose of a dosing regimen) and persistence (duration of time from initiation to discontinuation of the therapy) with oral osteoporosis medications are poor (Imaz et al., 2010; Hiligsmann et al., 2012a; Hiligsmann et al., 2012b; Fatoye et al., 2019). A recent observational study estimated that 53% of the study population achieved a medication possession ratio (MPR) of 80% or higher 6 months after initiating therapy, and the equivalent value for 7–12 months was only 43% (Kothawala et al., 2007). Persistence, or the length of time a patient continues therapy, is similarly poor. It has been reported that the rate of persistence among new users was 46% after 7–12-month treatment period (Kothawala et al., 2007).

Although poor compliance and persistence decrease the medicine costs, the effectiveness of treatment is also reduced, which reduces bone mineral density and in turn leads to a higher risk of fractures (Hiligsmann et al., 2010; Hiligsmann et al., 2012a; Hiligsmann et al., 2012b). Hence, in order to estimate the cost-effectiveness of the intervention in real-world settings, it is important that economic evaluations take compliance and persistence into account.

In our recent study (You et al., 2020), we demonstrated the cost-effectiveness of yearly intravenous zoledronic acid, which has a higher persistence and compliance rate. Further economic evaluation of alendronate is an evidence gap that could inform prescribers about the potential loss of benefits resulting from poor compliance and persistence. More specifically, we first compared the clinical and economic outcomes derived from a real-life setting with those expected with full compliance and persistence. In addition, we further evaluated the potential economic value of persistence-enhancing interventions.

## Methods

### Overview

The development of this model adhered to the recommendations for the conduct of economic evaluations in osteoporosis (Hiligsmann et al., 2019). We used an updated version of the previously validated individual-level state-transition model (You et al., 2020) to estimate the impact of the compliance and persistence on the cost-effectiveness of alendronate treatment for Chinese postmenopausal osteoporotic women aged 65 and older. The model estimated the outcomes including the number of fracture quality-adjusted life-years (QALYs); direct societal costs in 2018 US dollars (USD); and incremental cost-effectiveness ratios (ICERs) per QALY gained. Costs and health outcomes beyond the first year were discounted at an annual rate of 3%, which is consistent with Chinese guidelines for pharmacoeconomic evaluations (Liu, 2011). We assessed cost-effectiveness from the health care payer perspectives and considered three times per capita gross domestic product of China in 2018 (USD 29,340) as the threshold. We used TreeAge Pro 2018 (TreeAge Software Inc., Williamston, MA, USA) to perform our analyses.

### Model Structure

We modeled the disease progression of osteoporosis through six states: no fracture, hip fracture, clinical vertebral fracture, wrist fracture, other osteoporotic fractures, and death. The other osteoporotic fractures (i.e., humerus, distal forearm other than wrist, tibia/fibula, pelvis, or femur other than hip) as defined by the IOF-EFPIA report (Svedbom et al., 2013). The cycle length of the model was 1 year which was chosen to represent a clinically meaningful time interval. Each individual can sustain only one fracture per cycle and can experience up to two hip fractures but unlimited clinical vertebral, wrist, and other osteoporotic fractures during the entire study period. We used tracker variables to record individual characteristics and disease histories, which adjusted transition probabilities, costs, and utilities. Table 1 shows the key parameters used in the health economics model. A more detailed description of the model can be found in our previously published work (You et al., 2020).

**TABLE 1:** Summary of key parameters in the model.

Parameter	Value	Range	Distribution	References
Alendronate therapy				
Relative risk of hip fracture	0.45	0.27–0.68	Beta	Murad et al. (2012)
Relative risk of clinical vertebral fracture	0.50	0.33–0.79	Beta	Murad et al. (2012)
Relative risk of wrist fracture	0.50	0.34–0.73	Beta	Wells et al. (2008)
Relative risk of other fractures	0.78	0.66–0.92	Beta	Murad et al. (2012)
Persistence rate	0.57 (year 1)	N/A	N/A	Cheng et al. (2013)
Compliance rate	0.71 (year 1)	N/A	N/A	Cheng et al. (2013), Kishimoto and Maehara (2015)
Costs (2018 US dollars)				
Annual cost for alendronate	761.64	533.15–990.13	Triangular	National Development and Reform Commission (2019)
Hip fracture, direct costs	7103.25	4,972.28–9,234.23	Triangular	Qu et al. (2014)
Clinical vertebral fracture, direct costs	1,310.11	917.08–1,703.14	Triangular	Qu et al. (2014)
Wrist fracture, direct costs	967.34	677.14–1,257.54	Triangular	Qu et al. (2014)
Other fractures, direct costs	1,692.41	1,184.69–2,200.13	Triangular	Qu et al. (2014)
Annual cost for the posthip fracture	4,438.08	3,106.66–5,769.50	Triangular	Si et al. (2016)
DXA scan	85	59.5–110.5	Triangular	National Development and Reform Commission (2019)
Blood tests	72	50.4–93.6	Triangular	National Development and Reform Commission (2019)
Physician visit	10	7–13	Triangular	National Development and Reform Commission (2019)
Utilities				
Age 65–69	0.806	0.765–0.846	Beta	Sun et al. (2011)
Age 70–74	0.747	0.709–0.784	Beta	Sun et al. (2011)
Age 75–79	0.731	0.694–0.767	Beta	Sun et al. (2011)
Age 80–84	0.699	0.664–0.733	Beta	Sun et al. (2011)
Age 85+	0.676	0.642–0.709	Beta	Sun et al. (2011)
Hip fracture, first year (multiplier)	0.776	0.720–0.844	Beta	Si et al. (2014)
Hip fracture, subsequent year (multiplier)	0.855	0.800–0.909	Beta	Si et al. (2014)
Clinical vertebral fracture, first year (multiplier)	0.724	0.667–0.779	Beta	Si et al. (2014)
Clinical vertebral fracture, subsequent year (multiplier)	0.868	0.827–0.922	Beta	Si et al. (2014)
Wrist fracture (multiplier)	0.940	0.910–0.960	Beta	Hiligsmann et al. (2008)
Other fractures (multiplier)	0.910	0.880–0.940	Beta	Hiligsmann et al. (2008)
Annual fracture incidence per 1,000 persons (without intervention)				
Hip fracture, age 65–69	0.96	N/A	N/A	Wang et al. (2014)
Hip fracture, age 70–74	2.33	N/A	N/A	Wang et al. (2014)
Hip fracture, age 75–79	4.08	N/A	N/A	Wang et al. (2014)
Hip fracture, age 80–84	6.44	N/A	N/A	Wang et al. (2014)
Hip fracture, age 85+	6.59	N/A	N/A	Wang et al. (2014)
Clinical vertebral fracture, age 65–69	5.64	N/A	N/A	Bow et al. (2012)
Clinical vertebral fracture, age 70–74	8.74	N/A	N/A	Bow et al. (2012)
Clinical vertebral fracture, age 75–79	12.05	N/A	N/A	Bow et al. (2012)
Clinical vertebral fracture, age 80–84	21.19	N/A	N/A	Bow et al. (2012)
Clinical vertebral fracture, age 85+	26.89	N/A	N/A	Bow et al. (2012)
Wrist fracture, age 65–69	12.95	N/A	N/A	Lofthus et al. (2008)
Wrist fracture, age 70–74	13.17	N/A	N/A	Lofthus et al. (2008)
Wrist fracture, age 75–79	13.87	N/A	N/A	Lofthus et al. (2008)
Wrist fracture, age 80–84	15.01	N/A	N/A	Lofthus et al. (2008)
Wrist fracture, age 85+	15.10	N/A	N/A	Lofthus et al. (2008)
Other osteoporotic fractures, age 65–69	6.60	N/A	N/A	Mori et al. (2017a)
Other osteoporotic fractures, age 70–74	9.84	N/A	N/A	Mori et al. (2017a)
Other osteoporotic fractures, age 75–79	14.44	N/A	N/A	Mori et al. (2017a)
Other osteoporotic fractures, age 80–84	18.06	N/A	N/A	Mori et al. (2017a)
Other osteoporotic fractures, age 85+	26.06	N/A	N/A	Mori et al. (2017a)
Relative risks of fractures for individuals with osteoporosis				
Hip fracture, age 65–69	3.91	3.28–4.56	Gamma	Kanis et al. (2000), Johnell et al. (2005)
Hip fracture, age 70–74	3.13	2.80–3.47	Gamma	Kanis et al. (2000), Johnell et al. (2005)
Hip fracture, age 75–79	2.60	2.39–2.82	Gamma	Kanis et al. (2000), Johnell et al. (2005)
Hip fracture, age 80–84	2.04	1.91–2.17	Gamma	Kanis et al. (2000), Johnell et al. (2005)
Hip fracture, age 85+	1.92	1.78–2.05	Gamma	Kanis et al. (2000), Johnell et al. (2005)
Clinical vertebral fracture, age 65–69	2.59	1.19–4.27	Gamma	Marshall et al. (1996), Kanis et al. (2000)
Clinical vertebral fracture, age 70–79	2.15	1.15–3.15	Gamma	Marshall et al. (1996), Kanis et al. (2000)
Clinical vertebral fracture, age 80+	1.82	1.12–2.41	Gamma	Marshall et al. (1996), Kanis et al. (2000)
Wrist fracture, age 65–69	1.78	1.78–2.19	Gamma	Marshall et al. (1996), Kanis et al. (2000)
Wrist fracture, age 70–79	1.6	1.60–1.88	Gamma	Marshall et al. (1996), Kanis et al. (2000)
Wrist fracture, age 80+	1.45	1.45–1.64	Gamma	Marshall et al. (1996), Kanis et al. (2000)
Other osteoporotic fractures, age 65–69	2.19	1.78–2.59	Gamma	Marshall et al. (1996), Kanis et al. (2000)
Other osteoporotic fractures, age 70–79	1.88	1.60–2.15	Gamma	Marshall et al. (1996), Kanis et al. (2000)
Other osteoporotic fractures, age 80+	1.64	1.45–1.82	Gamma	Marshall et al. (1996), Kanis et al. (2000)
Annual mortality rate				
65–69	0.01031	N/A	N/A	Si et al. (2016)
70–74	0.02036	N/A	N/A	Si et al. (2016)
75–79	0.03784	N/A	N/A	Si et al. (2016)
80–84	0.06998	N/A	N/A	Si et al. (2016)
85+	0.13603	N/A	N/A	Si et al. (2016)
Excess mortality after a hip fracture				
Relative hazard for mortality within a year after a hip fracture	2.87	2.52–3.27	N/A	Haentjens et al. (2010)
Relative hazard for mortality for second and beyond after a hip fracture	1.73	1.56–1.90	N/A	Haentjens et al. (2010)
Proportion of excess mortality after a hip fracture directly attributable to a hip fracture	0.25	N/A	N/A	Kanis et al. (2003)
Discounts				
Costs	0.03	0–0.05	Triangular	Liu (2011)
Effectiveness	0.03	0–0.05	Triangular	Liu (2011)

### Fracture Incidence and Mortality Rates

Hip and vertebral fracture incidences were derived from reported epidemiological data in China (Bow et al., 2012; Wang et al., 2014). Estimation of the incidence rates of the wrist and other osteoporotic fractures in the Chinese context was not available; hence, we utilized data collected from an Asian population (Melton et al., 1999; Lofthus et al., 2008). The incidence of fracture in the general population was further adjusted to accurately reflect the fracture risks of women with osteoporosis. The method calculated the relative risks for bone mineral density using a method previously described (Marshall et al., 1996; Kanis et al., 2000; Johnell et al., 2005).

Baseline mortality rates for age-stratified Chinese women were retrieved from the China Public Health Statistical Yearbook (National Health Committee of the People's Republic of China, 2018) and increased mortality was assumed for individuals who experienced the hip fracture (Haentjens et al., 2010). Because excess mortality may be attributable to comorbidities in this older population, only 25% of the excess mortality was considered to be attributable to the fractures themselves (Kanis et al., 2003). There was no increase in mortality following clinical vertebral, wrist, and other fractures (Mori et al., 2017a; Mori et al., 2017b).

### Treatment

We assumed that treated women received alendronate 70 mg once a week for 5 years. Relative risks for fractures in women taking alendronate were based on the recent systematic reviews (Wells et al., 2008; Murad et al., 2012). It was assumed that reductions in fracture risk during therapy were consistent regardless of patients’ age and there was no significant change in bioequivalence between brand name and generic drugs. We also assigned the cost of one general consultation visit, bone mineral density, and biochemical test per year, as suggested by the Chinese guidelines for the diagnosis and treatment of primary osteoporosis (Chinese Society of Osteoporosis and Bone Mineral Research, 2019).

Inadequate medication compliance and persistence are known to be major problems in all patients with osteoporotic disease (Stevenson and Selby, 2014). We considered compliance and persistence rates of alendronate obtained on the observational studies in the Chinese or Asian population (Cheng et al., 2013; Kishimoto and Maehara, 2015). Compliance rates with oral alendronate were higher in clinical than observational studies. The influence of their difference was incorporated into the microsimulation model by assuming a linear relationship between the relative risk reduction and medication compliance (Mori et al., 2017a; Mori et al., 2017b). In addition, we modeled the residual effects of alendronate for those who discontinue therapy (called offset-time effect). We assumed that if individuals stopped treatment, they received no further therapy and offset time was assumed to be equal to their treatment period (Hiligsmann et al., 2012a; Hiligsmann et al., 2012b).

### Costs

The cost of alendronate was based on different brand prices and corresponding market share in China. Total medication costs were multiplied by their compliance and persistence levels. We charged the cost of 6-month alendronate supply for individuals who discontinued alendronate within the first year. The estimated annual costs related to hip fracture of the first year and long-term care costs were obtained from previously published studies in Chinese setting (Qu et al., 2014; Si et al., 2016). Costs of physician visits, DXA scan, laboratory tests, and nursing home residence were collected from the health system or the National Development and Reform Commission of China (National Development and Reform Commission, 2019). All original costs were converted to a common currency and price year, 2018 United States dollars (USD), given the latest version of a web-based cost converter (CCEMG-EPPI-Centre Cost Converter, 2008).

### Utilities

The Chinese National Health Services Survey in China has established the utility values in osteoporosis (Sun et al., 2011). No disutilities were assumed for simulated individuals without fractures. Fracture events were associated with decrements in utility values which differed between the fracture sites and time. The quality-of-life multipliers were based on a recent meta-analysis (Hiligsmann et al., 2008; Si et al., 2014).

### Model Simulation and Sensitivity Analysis

We performed base-case, deterministic (one-way) sensitivity, probabilistic sensitivity, and scenario analyses. For baseline analysis, we ran the model with 100,000 iterations (100,000 individuals through the model one at a time). One-way sensitivity analysis was undertaken to examine the effect of each key model parameter, including fracture costs and disutilities, medication costs, initial age of treatment, time horizon, residual effect, and discount rates. Probabilistic sensitivity analysis was conducted to evaluate the impact of the joint uncertainty surrounding the model variables using Monte-Carlo simulations (1,000 simulations and 10,000 trials per simulation). We also examined different scenarios: A) the individuals with full compliance, B) the individuals with full persistence, C) the individuals with both full persistence and full compliance, and D) potential persistence-enhancing interventions.

## Results

### Model Validation

The probability of dying by 105 years for untreated individuals at the ages of 65, 70, 75, and 80 predicted by our model was 99.0%, 98.8%, 98.5%, and 98.5%, respectively. Model-predicted mortality risks were comparable to the Chinese life table (National Health Committee of the People's Republic of China, 2018). We also projected that, without intervention, the cumulative probability of having at least one hip fracture or clinical vertebral fracture is equal to 11.099% and 39.693%, respectively, which is comparable to the epidemiological data in China (Chinese Society of Osteoporosis and Bone Mineral Research, 2019).

### Base-Case Findings

Table 2 presented the total health care costs, the number of fractures, QALYs, and ICER estimated by the model. Compared with no treatment (mean cost USD 9,411; mean effect 12.623 QALYs), alendronate treatment in the real-world setting (mean cost USD 10,149; mean effect 12.675 QALYs) was associated with an overall increase in total health care cost of USD 738 and in QALYs of 0.052, yielded in an ICER of USD 14,192.308/QALY gained. Besides, both NMB and NHB were positive, and further indicated oral alendronate is more cost-effective than no intervention.

**TABLE 2:** Results of base-case and scenario analyses.

	Different scenarios					Incremental values			
	NT	RW	FC	FP	FC + FP	RW vs. NT	FC vs. NT	FP vs. NT	FC + FP vs. NT
Patient cost over lifetime (2018 USD)									
Treatment cost	0	890	1,021	1,943	2,319	890	1,021	1,943	2,319
Total disease cost	9,411	9,254	8,670	7,907	7,667	–157	–741	–1,504	–1,744
Acute fracture cost	3,768	3,712	3,610	3,427	3,375	–56	–158	–341	–393
Long-term fracture cost	5,643	5,542	5,060	4,480	4,292	–101	–583	–1,163	–1,351
Total health care cost	9,411	10,149	9,707	9,850	9,987	738	296	439	576
Outcome over lifetime									
All fractures per patient	1.461	1.442	1.438	1.418	1.350	–0.019	–0.023	–0.043	–0.111
QALYs per patient	12.623	12.675	12.683	12.769	12.904	0.052	0.060	0.146	0.281
ICER	—	—	—	—	—	14,192.308	4,933.333	3,006.849	2,049.822
NHB	—	—	—	—	—	0.027	0.050	0.131	0.261
NMB	—	—	—	—	—	787.680	1,464.400	3,844.640	7,668.540

USD, United States dollars; QALYs, quality-adjusted life-years; ICER, incremental cost-effectiveness ratio; NHB, net health benefit; NMB, net monetary benefit; NT, no treatment; RW, real-world setting; FC, full compliance; FP, full persistence.

### Sensitivity Analysis Findings

Deterministic sensitivity analysis showed that the most impactful parameters in the model were the time horizon and the residual effect. The ICER was markedly increased to USD 994,000/QALY when reducing the time horizon from lifetime to 5 years. Assuming no residual effect following treatment resulted in ICER increase to USD 49,294.118/QALY (Table 3).

**TABLE 3:** Results of one-way analyses.

Parameter	Cost (2018 USD)		ΔC	Effectiveness (QALYs)		**Δ**E	ICER (USD/QALY gained)
	No treatment	Alendronate		No treatment	Alendronate		
Starting age of treatment: 80	4,816	5,283	467	5.351	5.400	0.049	9,530.612
Starting age of treatment: 75	6,483	7,003	520	7.321	7.368	0.047	11,063.830
Starting age of treatment: 70	8,076	8,651	575	9.631	9.675	0.044	13,068.182
5-year time horizon	604	1,598	994	3.860	3.870	0.010	99,400.000
No residual effect	9,422	10,260	838	12.649	12.666	0.017	49,294.118
Discount rate: 0	10,557	11,421	864	13.867	13.941	0.074	11,675.676
Discount rate: 0.05	8,407	9,032	625	11.661	11.692	0.031	20,161.290
Fracture costs 30% higher	12,319	12,808	489	12.654	12.681	0.027	18,111.111
Fracture costs 30% lower	6,605	7,329	724	12.647	12.686	0.039	18,564.103
Fracture disutilities 30% higher	9,461	10,098	637	12.888	12.925	0.037	17,216.216
Fracture disutilities 30% lower	9,542	10,176	634	12.438	12.474	0.036	17,611.111
Alen costs 30% higher	9,418	10,204	786	12.634	12.678	0.044	17,863.636
Alen costs 30% lower	9,457	9,925	468	12.641	12.679	0.038	12,315.789
Excess mortality 50% higher	11,647	12,273	626	12.917	12.945	0.028	22,357.143
Excess mortality 0%	8,819	9,483	664	12.571	12.604	0.033	20,121.212

**FIGURE 1:**
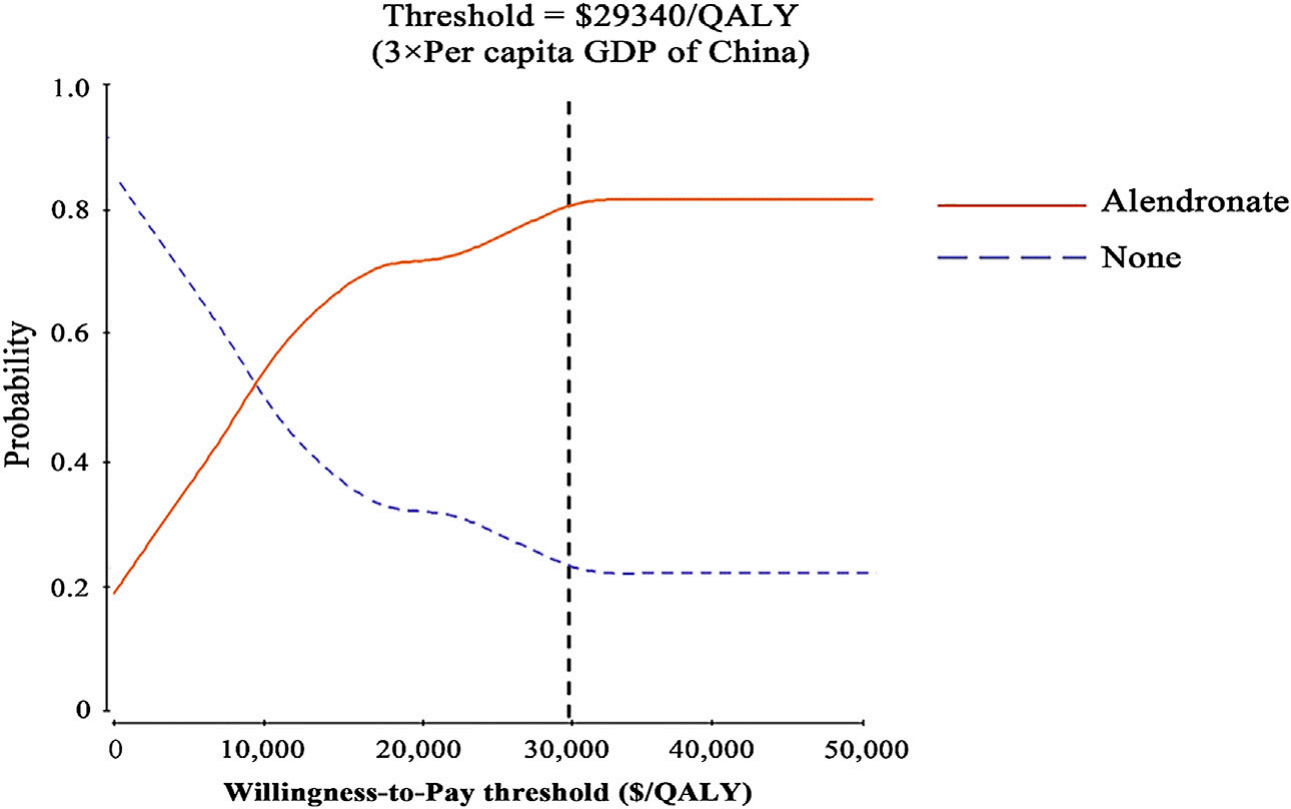
Results of probabilistic sensitivity analyses. The cost-effectiveness acceptability curves represent probabilities of being cost-effective achieved by the alendronate strategy compared to no treatment at thresholds for postmenopausal osteoporotic women.

Probabilistic sensitivity analysis confirmed the aforementioned results (Figure 1). At a threshold of USD 29,340/QALY, the probability that alendronate would be cost-effective was approximately 80% for individuals aged 65.

### Scenario Analysis Findings

**FIGURE 2:**
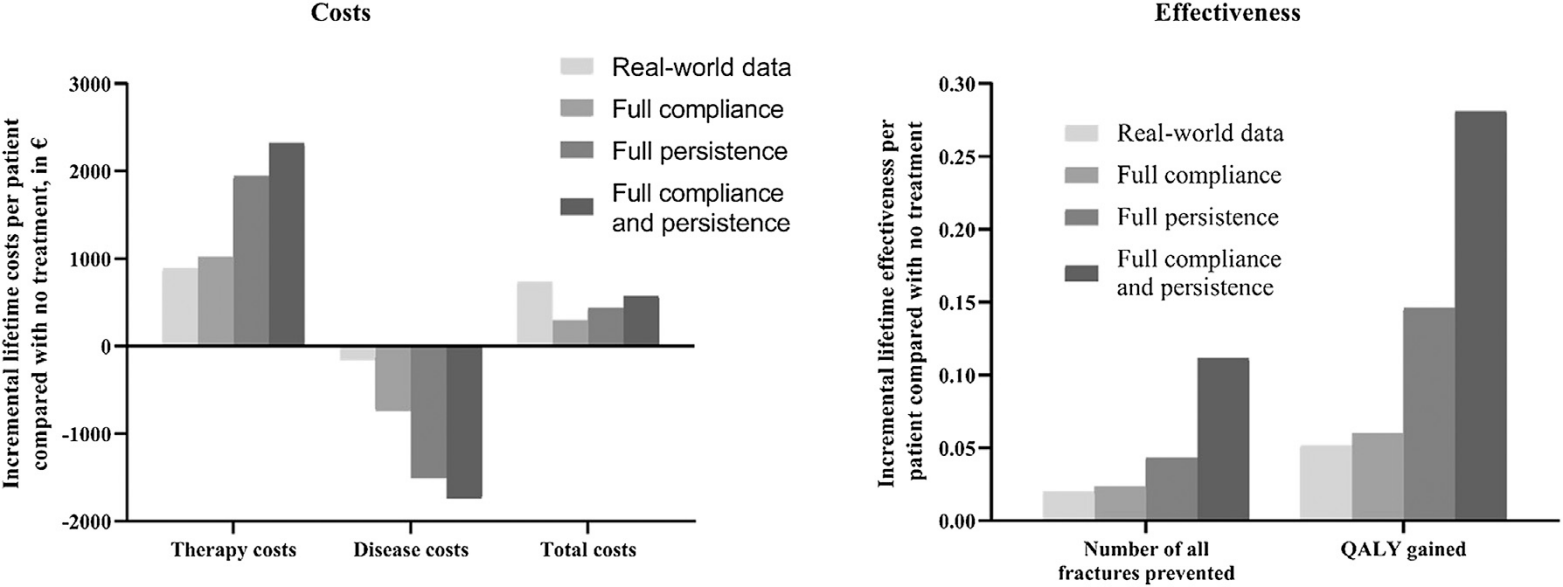
Impact of medication compliance and persistence on therapy, disease, total costs, and health outcomes (expressed as number of fractures prevented and QALY gained). QALY, quality-adjusted life-year.

The results of the scenario analysis considering alendronate therapy compliance and persistence were shown in Table 2 and Figure 2. The lifetime cost per person was USD 9,707 for the full compliance scenario, USD 9,850 for the full persistence scenario, and USD 9,987 for both full persistence and full compliance scenario. Total cost was lower in the scenario analysis than in the real-world setting, as the prevented costs of treating additional osteoporotic fractures resulting from noncompliance and persistence exceed the cost of the additional therapy induced by the improved compliance and persistence.

Effectiveness was measured as the number of all osteoporotic fractures and quality-adjusted life-years. The lifetime number of all fractures per person was 1.438 for the full compliance scenario, 1.418 for the full persistence, and 1.350 for both full compliance and full persistence. Hence, the number of osteoporotic fractures prevented in real-world setting represented 81.2%, 43.8%, and 17.1% to that estimated with full compliance, full persistence, and both full compliance and full persistence scenario, respectively. Mean lifetime QALYs were estimated at 12.683, 12.769, and 12.904 in all scenarios tested, respectively. The QALYs gained in the real-world scenario represent 86.7%, 35.6%, and 18.5% to that obtained under the above three scenarios, respectively.

Compared with no treatment, the ICER for the three scenarios ranged from USD 2019.822/QALY to USD 4933.333/QALY. These results were all lower than those derived from real-world analysis. It should be noted that three different scenarios were associated with lower costs and great QALYs than the real-world setting, indicating that the improvement of compliance and persistence was found to be cost-saving.

**FIGURE 3:**
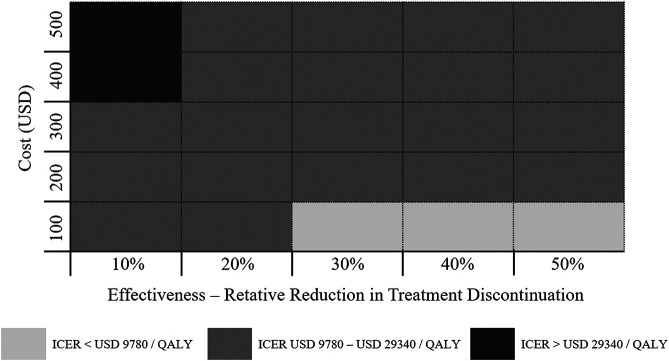
Economic value of persistence-enhancing interventions according to a given range of their costs and effectiveness values. Each block represents a possible intervention characterized by its cost and effectiveness. The color coding denotes the cost-effectiveness of the intervention.

Figure 3 displayed the economic assessment of persistence-enhancing interventions based on differential reduction in treatment discontinuation and their corresponding cost. When the reductions in treatment discontinuation were high (> 30%) and the invention costs were low (< USD 100), the ICER was less than USD 9,780/QALY (1 × GDP per capita) and could be considered highly cost-effective. Conversely, when the invention costs were high (> USD 400) and the reductions in treatment discontinuation were low (< 10%), the ICER was more than USD 29,340/QALY (3 × GDP per capita) and could be considered not cost-effective. For other potential combinations of values within the given range, the ICER between USD 9,780/QALY and USD 29,340/QALY is regarded as acceptable cost-effectiveness limit.

## Discussions

In this study, we used a modeling approach incorporating the medication compliance and persistence to examine the cost-effectiveness of oral alendronate treatment versus no intervention in the treatment of osteoporosis in Chinese postmenopausal women. Our base-case analysis revealed that, compared with no treatment, oral alendronate therapy 70 mg once a week for 5 years was a high-value treatment at a threshold of USD 29,340/QALY.

The key variable in the current research was the medication persistence and compliance. Although oral alendronates have been demonstrated to be high value with current medication discipline, they are more cost-effective with full compliance and persistence. In addition, persistence was found to have a greater impact on cost-effectiveness than compliance. Full persistence in our model would yield an ICER of USD 3,006.849/QALY, lower than the equivalent value for the full compliance (USD 4,933.333/QALY). It should be noted that this heightened persistence rate of oral alendronate was emphasized by our assumption of a residual effect from treatment; the risk for fracture returned to rates in the absence of therapy over the same years as the treatment duration in a gradual linear fashion after completing the therapy. This is also examined by deterministic sensitivity analyses, in which we assumed no residual effect after the treatment; the ICER of oral alendronate was sharply increased to USD 9,294.118/QALY. Hence, interventions to enhance persistence are necessary to decrease the considerable economic burden caused by the nonpersistence with oral alendronate.

Our results confirmed prior work that it is important to include medication persistence and compliance in pharmacoeconomic analysis of osteoporosis treatment. The two studies of Hiligsmann and colleagues (Hiligsmann et al., 2010; Hiligsmann et al., 2012a; Hiligsmann et al., 2012b) which were focused on oral bisphosphonates suggested that poor adherence with osteoporosis medications results in approximately a 50% reduction in the potential benefits observed in clinical trials and a doubling of the cost per QALY gained from these medications. Programs to improve compliance were considered to be an efficient use of resources. In contrast, the study of Chen and colleagues (Si et al., 2016) in the China setting which compared raloxifene treatment with conventional treatment (alendronate, calcitonin, and calcium combined with vitamin D) found opposite results. In this study, although high persistence and compliance increased both clinical effectiveness and average costs, the improvement in effectiveness was marginal in their research, thus resulting in higher ICER compared with the real-world scenario. The main reasons for such a difference could be attributed to the costs for fracture inpatients and the comparator.

In our previous study (You et al., 2020), in which we examined the cost-effectiveness of once-yearly injection of zoledronic acid compared with oral alendronate once a week for postmenopausal osteoporotic women without prior history of fracture in China, we concluded that zoledronic acid was cost-effective at all starting ages and even cost-saving in scenario analysis mainly based on zoledronic acid’s higher persistence leading to higher efficacy. In this study, we came to a similar conclusion that medication persistence plays a key role in shaping perceptions of fracture risk and osteoporosis drug effectiveness. In addition, we extend the prior work by designing a meaningful framework for assessing the economic value of persistence-enhancing interventions. We assessed the potential combination of the intervention costs from USD 100 to 500 and the relative reduction in discontinuation from 10% to 50%.

There are limitations associated with the current study. First, like all models, the generalizability of the results to the target population of other races/ethnicities or in other countries may be uncertain due to the heterogeneity of payer perspectives and the country-specific epidemiologic data used. Moreover, although much of the data which constructed the model were obtained from the Chinese context, some data were also extrapolated from other countries. An updated pharmacoeconomic analysis should be explored when these data are available in Chinese setting. Second, compliance and persistence rates were derived from a retrospective study (Cheng et al., 2013) in which whether patients actually took the dispensed drug is unknown. The study assumed that patients who obtain prescription refills do take their medications based on chart review. As a result, compliance may be overestimated. Third, our analysis did not examine the impact of restart therapy after discontinuation. We assumed that those who did not take alendronate continued not to take the medication in this model, which may not always mimic treatment in the real world because some patients might return to treatment after this period. Finally, we did not perform a budget impact analysis to assess the potential cost-savings of this strategy. Due to the enormous amount of osteoporosis cases in China, the financial burdens for the health care system might be heavy.

Despite these limitations, our research has several key strengths. First, to the best of our knowledge, this is the first pharmacoeconomic analysis that compared oral alendronate to no treatment in a Chinese population. Second, we incorporated medication persistence and compliance, which are considered to be critical impedance to osteoporosis management, into our hybrid modeling and extensively examined how these changes in parameters have an impact on model results. We further assessed the potential cost-effectiveness of persistence-enhancing interventions according to a given range of their costs and effectiveness values.

In conclusion, oral alendronate is considered to be a high-value therapy option for postmenopausal osteoporotic women from the perspective of Chinese health care payers, and further interventions to improve osteoporosis medication persistence will likely have favorable ICERs.

## Data Availability Statement

The raw data supporting the conclusions of this manuscript will be made available by the authors, without undue reservation, to any qualified researcher.

## Author Contributions

ZL conceived and designed the research. RY collected and analyzed the data. All authors wrote the manuscript.

## Funding

This research did not receive any specific grant from any funding agency in the public, commercial or not-for-profit sector.

## Conflict of Interest

The authors declare that the research was conducted in the absence of any commercial or financial relationships that could be construed as a potential conflict of interest.
